# Attitudes towards suicidal behaviour in outpatient clinics among mental health professionals in Oslo

**DOI:** 10.1186/1471-244X-13-90

**Published:** 2013-03-19

**Authors:** Astrid Berge Norheim, Tine Kristin Grimholt, Øivind Ekeberg

**Affiliations:** 1 Diakonhjemmet hospital, Postboks 23, Vinderen 0319, Oslo; 2 Regional Centre of Violence Traumatic stress and suicide Prevention Eastern Norway, Gaustad, Oslo University Hospital, Gaustad 0514, Oslo; 3 Department of Acute Medicine, Oslo University Hospital Ullevål, Postboks 4956, Nydalen 0424, Oslo

**Keywords:** Attitudes, Suicide, Suicidal behaviour, Suicide ideation, Health professionals

## Abstract

**Background:**

To investigate attitudes of professionals working in mental health care outpatient clinics in Child and Adolescent Psychiatry (CAP) (for children and adolescents aged 0–18 years) and District Psychiatric Centres (DPC) (for adults aged 18–67 years).

**Methods:**

Professionals in four outpatient units in Oslo were enrolled (n = 229: 77%). The Understanding of Suicidal Patient scale (USP) (11 = positive to 55 = negative) and Attitudes Towards Suicide questionnaire (ATTS) (1 = totally disagree to 5 = totally agree) were used to assess professionals’ attitudes. Questions explored competence, religion, experiences of and views on suicidal behaviour and its treatment.

**Results:**

All the professionals indicated positive attitudes (USP 18.7) and endorsed the view that suicide was preventable (ATTS 4.3). Professionals who had received supervision or were specialists had attitudes that were more positive. Professionals in CAP were less satisfied with available treatment. Psychiatric disorders were considered the most common cause of suicidal behaviour, and psychotherapy the most appropriate form of treatment. The professionals confirmed that patients with other disorders of comparable severity are followed up more systematically.

**Conclusions:**

The professionals showed positive attitudes with minor differences between CAP and DPC.

## Background

Suicide is one of the most frequent causes of death in young age groups and represents a serious health problem affecting patients, families and societies worldwide [1]. It is a complex problem that is often associated with mental illness [2]. Suicidal ideation and behaviour are risk factors for suicide and are important triggers for mental health treatment [2,3]. The professional’s priorities and attitudes towards suicidal patients are important in motivating patients to engage in treatment and manage suicidal indications. Many suicidal patients are vulnerable and may feel rejected easily. Suicidal behaviour indicates a significant health problem that requires professionals to convey appropriate attitudes towards their patients to achieve effective treatment. Attitudes affect emotions, cognition and behaviour. However, the topic has attracted relatively limited systematic research interest and documented interventions have been limited, which may partly reflect attitudes towards suicide [4] and its low status in health care [5].

The capacity to treat patients with suicidal behaviour has increased in mental health outpatient units such as Child and Adolescent Psychiatry (CAP) for patients aged 0–18 years and District Psychiatric Centres (DPC) for patients aged 18–67 years [6]. Studies from different cultures indicate that attitudes can influence referring to aftercare following a suicide attempt [7]. We do not know if the quality of care offered to patients with suicidal behaviour is the same as the care offered to patients with other severe health problems, or if the age of the patient influences professionals’ attitudes [8] and their ability to treat. The fact that suicide is more common among adults [9] while self-harm and suicidal behaviour is more common among adolescents [10] might be the reason why suicidal behaviour is more serious in adults.

Studies from other health-care fields show that professionals report more irritation, anger, frustration and helplessness towards patients who self-harm than towards other patients [8]. Patients who self-harm describe feeling humiliated by the experience of receiving physical treatment delivered without empathy, which differentiated them from patients with other conditions. Staffs often lack knowledge about suicidal behaviour and ideation, and communication between patients and staff is perceived as poor. Patient feedback indicates a need for improvements in psychological assessment and aftercare [11].

Most studies on professionals’ attitudes are conducted in somatic [8,12,13] or psychiatric care in-patient units [8,14]. Attitudes towards suicidal patients differ within and between departments and professional groups. The most empathic attitudes are found among trained psychiatric professionals, professionals with experience, and those who have received supervision or have educational qualifications or special status [10]. The majority of studies have included nurses and physicians, with a relative minority of studies including psychologists and social workers. We have less knowledge of professionals’ attitudes towards suicidal behaviour in outpatient clinics. In these settings, they tend to work alone and with less control over their patients’ behaviour. At the same time as they have more independent responsibility compared with their in-patient unit counterparts, only physicians and psychologists have more formal independent responsibilities while other professional groups work by delegation [15]. The stresses of working with suicidal behaviour and the emotional burden of losing a patient [16,17] might also influence professionals’ attitudes.

For professionals generally, coping with accidents, illness and death in children and adolescents is more demanding than coping with the same scenarios involving adults. Professionals almost certainly experience a similar burden of coping with suicide in younger patients that may influence their attitudes [18]. Therefore, it is interesting to study whether attitudes towards suicide may differ between professionals in CAP and DPC. With an understanding that professionals in mental health outpatient units in Norway are at the forefront of suicide prevention efforts [6], we investigate the following questions.

### Aims

1. What is the range of attitudes towards suicidal patients among mental health professionals in Child and Adolescent Psychiatry (CAP) and the District Psychiatric Centre (DPC) outpatient units in Oslo, Norway?

2. Do attitudes differ according to profession, gender, age or religion?

3. Do experience, competence and understanding of suicidal behaviour vary by work site or profession?

## Methods

### Subjects

This study collected data by anonymous questionnaire from four CAP and four DPC outpatient units in Oslo. The units were selected to obtain a general picture of the socio-economic status of the four health regions in Oslo. The heads of all departments were asked to facilitate the study by distributing the questionnaires through internal mail and during staff meetings encouraging staff to participate. The paper questionnaires were completed anonymously and then each was labelled with the working place and a number. The inclusion period was from November 2010 to February 2011.

The response rate for DPC was 75% (122/162) and 79% for CAP (107/135). Age was recorded by 10-year intervals from 1 (under 30) to 5 (over 60) to help preserve anonymity.

As shown in Table 1, 65% of the professionals were women, with no significant differences in age between CAP and DPC. The age distribution was: below 30 (16), 30–39 (78), 40–49 (51), 50–59 (46), over 60 (35), no response (3). Psychologists (46%) were the largest professional group. “Others” (13%) was a group of various professions comprising mostly specialist teachers mainly employed in CAP. All nurses (15%) were employed in CAP. Comparisons between working place were conducted both with and without nurses.

**Table 1:** Characteristics of professions in CAP and DPC

	**CAPn = 105–107n (%)**	**DPCn = 221–122n (%)**	**Totaln = 226–229n (%)**	**p**
*Profession*				
Physicians	14 (13)	19 (15)	33 (15)	n.s.
Psychologists	54 (51)	52 (43)	106 (46)	n.s.
Nurses	0	34 (28)	34 (15)	<0.001
Social workers	15 (14)	11 (9)	26 (11)	n.s.
Other	24 (22)	6 (5)	30 (13)	<0.001
*Gender*				
Women	71 (68)	76 (63)	147 (65)	n.s.
Men	34 (33)	45 (37)	79 (35)	
Religion				
Christian	63 (59)	74 (61)	137 (60)	n.s.
No religion	39 (37)	47 (39)	86 (37)	n.s.
Other religion	5 (5)	1 (1)	6 (3)	n.s.
*Age*				
30 years	5 (5)	11 (9)	16 (7)	n.s.
31–40 years	40 (38)	38 (31)	78 (35)	n.s.
41–50 years	22 (21)	29 (24)	51 (23)	n.s.
50 years	38 (36)	43 (36)	81 (36)	n.s.

n.s.: Not significant CAP: Child and Adolescent Psychiatry. DPC: District Psychiatric Centres.

The religious backgrounds reported by professionals were 60% Christian, 37% no religion, and 3% other religion. The groups with no/other religion were too small for analysis.

### Assessments

The Understanding of Suicidal Patients scale (USP) was used. The last 11 of the 14 items in the questionnaire from the final study are used to create the USP scale.

Responses are recorded on a scale from 1 (completely agree) to 5 (completely disagree) [14]. Three of the items were reversed. A sum score was calculated from 11 (positive) to 55 (negative) as a measure of understanding and willingness to help suicidal patients. Cronbach’s alpha was 0.56. Scores lower than 18 to 22 were considered positive [12,13].

The ATTS scale consists of 37 items on a five-point scale from 1 (disagree completely) to 5 (agree completely). It was developed from the Suicide Opinion Questionnaire (SOQ) to measure broad dimensions of attitudes towards such items as suicide as a right, comprehensibility, communication, preventability and taboos [19]. The dimensions are found by factor analysis. Although the questionnaire has been criticized for the generality of its dimensions, it has relatively high reliability [20] and has been used in different populations and cultures [19,21,22].

In the present study, varimax rotation and Kaiser normalization were conducted. Based on the scree plot, eigenvalues, and analysis results, we chose a 4-factor solution for analyses.

Professionals were asked about their experiences with patients who committed suicide, attempted suicide or self-harmed. Furthermore, they were asked to self-assess their competence in the form of education or supervision and whether they used written guidelines for treating suicidal patients.

The professionals were asked if the views they expressed were informed by their religious background. They were asked about what they considered important in treatment, their level of satisfaction with available treatment, and whether they considered a patient death by suicide as a professional failure. Items on views on suicidal issues and treatment were recorded on a five-point scale where 0 referred to “disagree” and 4 referred to “agree”.

### Statistics

Data are presented as means with 95% confidence intervals (CI). The level of significance was set at p < 0.05. The English version of SPSS, version 19, was used to conduct Chi-square, Student’s *t* tests, ANOVA, and factor analyses with varimax rotation.

### Ethics

The Regional Ethics Committee for South Eastern Norway determined that the study needed only to be evaluated by the Data Protection Officer at Oslo University Hospital, who approved the study. Data were collected anonymously.

## Results

### Attitudes in child and adolescence (CAP) and adult psychiatry (DPC) professionals

The factor analysis of ATTS initially suggested a 12-factor solution explaining 62% of the variance. The scree plot, eigenvalues, and factor loadings showed a 4-factor structure that explained 35% of the variance.

Factor 1 showed an eight-item structure with items 5, 16, 18, 20, 29, 32, 34, and 36. The factor explained 15% of the variance. The factor loadings were between 0.77 and 0.52 and the Cronbach’s alpha was 0.84. The factor reflects accepting attitudes towards suicide such as item 5: “Suicide should be accepted as a way to shorten an incurable illness”.

Factor 2 showed a seven-item structure with the items 6, 7, 11, 12, 21, 23, and 33. The factor explained 10% of the variance. The factor loadings were between 0.63 and 0.49 and Cronbach’s alpha was 0.66. This factor reflects attitudes such as “Suicide should not be talked about and cannot be prevented”, as measured by item 11: “There is a risk of suicidal thoughts developing in a person if they are asked about their thoughts on suicide”.

Factors 3 and 4 were excluded because of their low Cronbach’s alpha scores.

As shown in Table 2, the attitudes towards patients with suicidal behaviour were quite positive with scores below 22. The professionals in CAP scored 19.2 and in DPC 18.3 (ns*). Differences between professional groups were not significant in CAP or DPC nor were there significant differences when nurses were excluded or included.

**Table 2:** Willing and understanding attitudes towards suicidal patients (USP)

	**CAPn = 100Mean (95% CI)**	**DPCn = 120Mean (95% CI)**	**Totaln = 220Mean (95% CI)**	**p**
Women (n = 140)	19.4 (18.3–20.6)	18.1 (17.2–19.0)	18.7 (17.7–19.2)	0.065
Men (n = 77)	18.8 (17.4–20.2)	18.6 (17.4–19.8)	18.7 (17.8–19.6)	0.848
Physicians (n = 32)	18.9 (15.9–21.8)	18.9 (17.0–20.8)	18.9 (17.3–20.4)	0.976
Psychologists (n = 103)	18.5 (17.4–19.7)	18. 3 (17.2–19.4)	18.4 (17.6–19.2)	0.802
Nurses (n = 33)	*	18.1 (16.8–19.4)	18.1 (16.8–19.4)	*
Social workers (n = 23)	20.5 (17.6–23.4)	18.5 (16.4–20.6)	19.6 (17.8–21.4)	0.269
Others (n = 29)	20.2 (18.1–22.3)	17.1 (13.3–21.1)**	19.6 (17.8–21.4)	0.163
Total (n = 220)	19.2 (18.3–20.1)	18.3 (17.6–19.0)	18.7 (18.2–19.3)	0.108

Understanding of Suicidal Patient Scale (USP). CAP: Child and Adolescent Psychiatry. DPC: District Psychiatric Centres. * No nurses are employed in CAP. ** Too few to consider. Scale: 11 = positive to 55 = negative. ns = not significant.

There was no significant difference between CAP and DPC (mean 2.6 vs. 2.5, respectively) in factor 1 (suicide is acceptable). However, physicians in CAP and DPC (mean 2.3) considered suicide less acceptable than did psychologists or social workers (mean 2.7 vs. 2.8, respectively; p < 0.001). Only the DPC nurses had the same attitude as physicians (mean 2.2). There was also a significant difference in factor 1 between professionals who were Christian and those who had no religion (mean 2.5 vs. 2.8, respectively, p < 0.001).

There was no significant difference between CAP and DPC (mean 2.3 vs. 2.2, respectively, p = 0.441) in factor 2 (suicide should not be talked about and cannot be prevented) (Table 3). Significance was borderline for professional groups in CAP (p = 0.025). There were no significant differences in factors 1 and 2 according to age or gender.

**Table 3:** Factors according to CAP and DPC (ATTS)

	**CAPn = 107Mean (95% CI)**	**DPCn = 122Mean (95% CI)**	**Totaln = 229Mean (95% CI)**
**Factor 1: Suicide is acceptable**	2.6 (2.5–2.7)	2.4 (2.3–2.5)	2.5 (2.4–2.6)
Physicians	2.3 (2.1–2.6)	2.2 (2.0–2.5)	2.3 (2.1–2.4)
Nurses	*	2.2 (2.0–2.4)	2.2 (2.0–2.4)
Psychologists	2.7 (2.5–2.8)	2.7 (2.5–2.8)	2.7 (2.6–2.8)
Social workers	2.8 (2.4–3.1)	2.7 (2.5–2.9)	2.7 (2.4–2.9)
Other	2.4 (2.2–2.6)	2.2 (1.6–2.7)**	2.3 (2.2–2.3)
Between professions	p = 0.035	p < 0.001	p < 0.001
Christians	2.6 (2.5–2.8)	2.3 (2.2–2.4)	2.5 (2.4–2.6)
No religion	2.7 (2.5–2.9)	2.9 (2.7–3.0)	2.8 (2.7–2.9)
Between religion	p = 0.589	p < 0.001	p < 0.001
**Factor 2: Suicide should not be talked about and cannot be prevented**	2.3 (2.2–2.3)	2.2 (2.1–2.3)	2.2 (2.2–2.3)
Physicians	2.2 (2.0–2.4)	2.2 (2.0–2.5)	2.2 (2.1–2.3)
Nurses	*	2.2 (2.1–2.4)	2.2 (2.1–2.4)
Psychologists	2.2 (2.1–2.3)	2.2 (2.1–2.3)	2.2 (2.1–2.2)
Social workers	2.4 (2.1–2.6)	2.3 (2.0–2.5)	2.3 (2.3–2.5)
Other	2.5 (2.2–2.7)	2.4 (1.9–2.9)**	2.4 (2.2–2.6)
Between professions	p = 0.026	p = 0.976	p = 0.025
Christians	2.3 (2.2–2.4)	2.2 (2.2–2.4)	2.2 (2.2–2.4)
No religion	2.3 (2.2–2.6)	2.3 (2.2–2.5)	2.3 (2.3–2.5)
Between religions	p = 0.497	p = 0.317	p = 0.238

Scale: 1, “completely disagree” to 5, “completely agree”. * No nurses are employed in CAP. ** Too few to consider. CAP: Child and Adolescent Psychiatry. DPC: District Psychiatric Centres.

Specialists (psychiatrist, clinical psychologist or psychiatric nurse) had less accepting attitudes to suicide than non-specialists (mean 2.3 vs. 2.6, respectively, p < 0.001).

Several of the separate ATTS items showed no significant differences between CAP and DPC professionals in their agreement that suicide could be prevented. The total scores for all professionals on the relevant items were: item 1, “It is always possible to help a person with suicidal thoughts” (mean 3.9); item 37, “Suicide can be prevented” (mean 4.3); item 9, “It is a humane duty to try to stop someone from dying by suicide” (mean 4.6); and item 24, “If someone wants to commit suicide, it is their business and we should not interfere” (mean 1.4).

There was no significant difference in religious background between CAP and DPC professionals (Table 2). The majority (88%) considered their religious background to impact not at all, or to a low degree, on their view of suicide.

### Experiences with suicidal patients, competence, written guidelines, and understanding of reasons for suicidal behaviour

#### Experience

**Figure 1:**
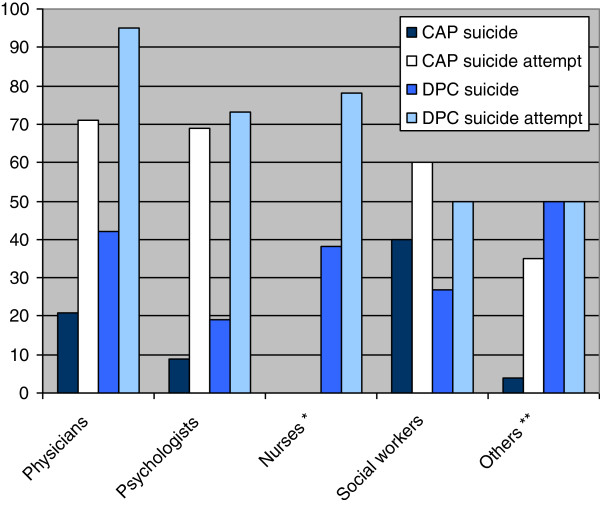
**Experience with suicide and suicide attempt in own patients %.** * No nurses are employed in CAP. ** There were only six “others” in DPC.

Experience of a patient suicide was 14% in CAP and 31% in DPC (p < 0.001). Further, 60% of those in CAP and 75% of those in DPC had experienced patients attempting suicide (p < 0.001) and 88% in CAP and 96% in DPC had experienced patients deliberately self-harming without suicidal intention (p = 0.005) (Figure 1).

#### Courses, supervision and written guidelines

A majority (80%) of professionals had participated in courses in suicide prevention. There were no significant differences by gender, working place or profession.

Systematic supervision with a focus on suicidal behaviour had been received by 64% in CAP and 81% in DPC (p < 0.001). Those who had received supervision reported more positive attitudes than those who had not (mean 15.5 vs. 21.0, respectively, p = 0.001), and were less inclined to accept suicide (ATTS factor 1, mean 2.4 vs. 2.8, respectively, p = 0.005). Written guidelines for suicide prevention were more common in DPC (95%) than in CAP (83%) (p < 0.05).

#### Views on causes of suicidal behaviour

As shown in Table 4, psychiatric disorder was considered the most important cause of suicide (mean 3.4) and biological changes in the brain (mean 1.9) of least importance. There were no significant differences between CAP and DPC in views on causes of suicide.

**Table 4:** **View on suicidal issues and treatment, scale 0–4**^**†**^

	**CAP n = 102–107 Mean (95% CI)**	**DPC n = 117–122 Mean (95% CI)**	**p**
**Causes of suicide**			
Psychiatric disorder	3.4 (3.3–3.5)	3.4 (3.3–3.5)	0.856
Inner turmoil and stress	2.9 (2.7–3.1)	2.7 (2.5–2.9)	0.123
Problems in the family	2.8 (2.7–3.0)	2.7 (2.6–2.8)	0.170
Use of alcohol	2.7 (2.5–2.9)	2.8 (2.7–3.0)	0.219
Biological changes in the brain	1.9 (1.7–2.0)	1.8 (1.7–2.0)	0.594
**Importance in treatment**			
Psychotherapy	3.5 (3.3–3.6)	3.3 (3.1–3.4)	0.039
Sleep and rest	2.9 (2.7–3.1)	2.9 (2.8–3.1)	0.867
Psychiatric in-patient treatment	2.7 (2.5–2.9)	2.8 (2.6–2.9)	0.566
Use of medication	2.4 (2.3–2.5)	2.8 (2.7–3.0)	< 0.001
Family therapy	2.7 (2.5–2.8)	2.5 (2.4–2.7)	0.138
Talk with priest/imam	2.0 (1.9–2.2)	2.2 (2.0–2.3)	0.254
Electroconvulsive therapy	1.3 (1.1–1.5)	1.4 (1.3–1.6)	0.286
**Satisfaction with treatment**			
Opportunity for hospitalization if needed	2.2 (2.0–2.4)	2.6 (2.5–2.8)	< 0.001
Adequate follow-up	1.9 (1.7–2.1)	2.2 (2.1–2.4)	0.008
Follow-up as good as for patients with heart disease	1.5 (1.3–1.7)	1.7 (1.6–1.9)	0.007
The suicide of a patient is a professional failure	1.6 (1.4–1.7)	1.3 (1.2–1.4)	0.008

CAP: Child and Adolescent Psychiatry. DPC: District Psychiatric Centres.

^**†**^ Scale: 0 refers to “totally disagree” and 4 refers to “totally agree”.

#### Importance for treatment

Psychotherapy was considered the most important treatment for suicidal behaviour in CAP and DPC (mean 3.5 vs. 3.3, respectively, p < 0.05) (Table 3). Psychologists (mean 3.6) indicated it significantly (p < 0.05) more important than physicians (mean 3.2), nurses (mean 3.1) and others (mean 3.1). Social workers’ responses (mean 3.4) fell halfway on the range of responses. Use of medication was considered more important in DPC than in CAP (mean 2.8 vs. 2.4, respectively, p < 0.001). Neither group considered electroconvulsive therapy (ECT) to be important.

#### Satisfaction with treatment

Table 4 shows that the professionals in CAP were generally less satisfied with available treatments than those in DPC. Both professionals in CAP and DPC (mean 1.9 vs. 2.2, respectively) only moderately considered the duration of follow-up to be sufficient. Clearly, neither group considered follow-ups to be of the same quality as follow-ups for patients with equally serious conditions such as heart disease.

#### Views of responsibility

Professionals in CAP (mean 1.6) agreed more with the statement “it is a professional failure if a patient commits suicide” than those in DPC (mean 1.3) (Table 4).

## Discussion

### Attitudes measured by USP and ATTS

The main findings of the present study were that professionals’ attitudes towards suicidal patients were generally positive and optimistic among professionals in the outpatient psychiatric units in Oslo and did not differ much between subgroups. Psychological treatment was considered most important, but the treatment facilities were only considered moderately sufficient, and not as efficient as for other groups with equally serious conditions such as heart disease.

The finding of positive attitudes is consistent with earlier studies, where the most empathic attitudes were found among professionals trained in psychiatry [8]. The positive findings from USP [14] in the present study were confirmed by ATTS [19]. This consistency in professionals’ beliefs that suicide can be prevented represents the optimism and hope that are important in treating suicidal patients. Our data show that professional specialty and supervision were associated with more understanding of patients and willingness to respond to suicidal behaviour, and less with acceptance of suicide as an outcome. These associations accord with other studies [8].

In the present study, we undertook a factor analysis of ATTS with two main factors: “Suicide is acceptable” and “Suicide should not be talked about and cannot be prevented”. These factors have also been found in other studies [21,22].

Views on the right to commit suicide differed somewhat between groups [23]. In the present study, the majority disagreed or was doubtful about “Acceptance of suicide”, with no significant difference between CAP and DPC. A recent study of adolescents in Slovenia showed that “Suicide is acceptable” was positively related to increased suicidal behaviour in adolescents [21], which is also in accordance with other findings [24]. Nevertheless, cultural differences may be at play. A study of the general population in Russia showed that men and women who reported earlier suicidal expressions were more non-accepting and condemning than persons who did not, whereas the opposite pattern was disclosed in Norwegian men and women and Swedish men [22].

The factor “Suicide should not be talked about and cannot be prevented” is often found in analyses of ATTS [21,22]. It is a factor that also represents the idea that one can initiate suicidal ideation by talking about suicide. Although threats of suicide can comprise communications in personal relations, it is more common that suicidal ideation is associated with depression, pain and shame, and talking about the problem is an important way to break introspection and deal with the underlying problem.

In Norway, the national mental health guidelines strongly recommend striving to identify suicidal risk [25]. In the present study, most professionals disagreed with “Suicide should not be talked about and cannot be prevented” (mean 2.2). The competency of all professionals to talk about suicidal ideation with their patients is important for mental health care. The small differences between CAP and DPC could reflect the higher relevance of the question when talking about suicide to those in CAP, who work mostly with the youngest children.

Overall, there does not seem to be a significant difference between professionals in DPC and CAP in preparedness for and security in working in suicide prevention. Professionals in CAP agreed somewhat more than in DPC with the statement “An academic failure occurs if a patient dies by suicide” (Table 4). This apparent attitude may reflect more worrying and negative attitudes towards youths than adults in psychiatric units [18]. It may also reflect the more demanding aspects of experiencing suicide among adolescents compared with adults, which may induce more feelings of failure and guilt.

### Attitudes according to professional background, gender, age and religious background

#### Professional background

The greatest differences between professions were found in the attitude “Suicide is acceptable”, where the majority (82%) of the professionals scored between 2.2 and 2.8. Physicians and nurses agreed less with the statement than the psychologists and social workers. The differences were also significant between the specialists, although the specialists were less likely to accept suicide.

Several explanations are possible for these differences. It may be because physicians and nurses are trained to work more with physical and mental illness while psychologists and social workers are trained to work more with psychological and social problems, which are not necessarily defined as illnesses.

Although there is no evidence that differing attitudes of professionals towards “acceptance of suicide” have any clinical impacts on suicidal prevention, reflection on how one’s own attitudes influence clinical work might be useful, especially in relation to physical and mental illness and pain. Reflection might be particularly important among psychologists and physicians who have more responsibility for assessing suicidal risk treatment for patients.

#### Gender and age

Although earlier studies found women to be more empathic than men towards suicidal patients [14], these findings could be connected to strong gender-role associations from which conclusions are difficult to draw [8]. Furthermore, it is unclear if age has an impact on attitudes; it is suggested that exposure to individuals who self-harm appears to have a greater impact [10]. In this study, we found only minor differences in attitudes according to gender and age.

#### Religiosity and attitudes

Religious background tends to impact on attitudes towards suicide. Durkheim found a correlation between religiosity and suicide in nineteenth-century Europe [24]. Many religions portray suicide as sinful. Suicide has been forbidden in many countries, and in Norway, burial of suicide victims in consecrated ground was forbidden until 1742 [26].

In this study, we asked professionals to consider the influence of their religious background on their views of suicide. The majority (88%) indicated it had little or no influence. Nevertheless, we found that professionals with a Christian background agreed significantly less with the statement that “suicide is acceptable” than professionals with no religious background. The study suggests that religious background continues to have some impact on attitudes, even though there seem to be small differences in ethical standards between Christian and non-religious people in Norway. Nevertheless, the study reveals that attitudes towards suicidal behaviour are influenced more by professional ethics than by religious background.

### Differences in experience, competence and view of suicidal behaviour and treatment

#### Suicide in own patients

It is expected that “suicide in own patients” will differ according to societies and cultures, and between working places and professionals. Studies show great differences between groups of professionals in their experiences of own patients’ suicide, but they all show that the suicide of a patient during the course of their treatment is stressful for professionals. The death of a patient during treatment is often associated with guilt and feeling insufficiently skilled. Avoidance of suicidal patients is described as a protective attitude [7]. Professionals who are treating patients with suicidal behaviour are more exposed to suicide in their own patients than professionals who are not providing such treatment.

In our study, 14% of the professionals in CAP and 31% in DPC reported having lost a patient to suicide (Figure 1). The difference according to age of the patient was as expected from statistics [8]. The professionals in DPC also had more experience with suicide attempts and self-harm than professionals in CAP. Suicidal gestures and deliberate self-harm without suicidal intention are common among adolescents [9]. A US study shows that a substantial proportion of young people who deliberately self-harm do not receive emergency mental health assessments and are discharged to the community without follow-up outpatient mental health care [7]. We do not yet know the magnitude of this issue in Norway.

Social workers in CAP and nurses in DPC had the most experience with suicidal patients, and psychologists the least. The number of social workers (n = 15) in CAP was small, so the finding calls for some reservations. In addition, the psychologists were younger and therefore had fewer years of experience. These findings therefore need confirmation through further studies.

### Competence

#### Supervision and courses in suicide prevention

Working with patients at risk of suicide is demanding and many studies have found that education and supervision can improve attitudes towards suicidal patients [8]. In our study, 80% of the professionals had participated in educational courses on suicidal prevention with no significant differences between CAP and DPC or between the professional groups. Knowledge about assessment is highlighted in the national guidelines [25]. This may have led to increased participation in professional education on suicide. However, there were no differences in attitudes between professionals who had participated in courses and those who had not.

Professionals in DPC reported having significantly more local guidelines than CAP and receiving more systematic supervision on suicidal patients. The study shows that access to supervision is associated with a more positive attitude.

### Mental illness and suicide

On the question of conceivable causes of suicide, there was general agreement that mental illness was most important and biological factors were less than moderately important causes of suicide. The finding is consistent with a general perception that suicide is strongly associated with mental illness [2,24,26]. However, the majority of psychiatric patients do not commit suicide, and some people who commit suicide do not have a history of mental illness. The strength of connections between mental illness and suicide has been questioned recently and the possibility for both over- and underestimation of mental disorders in suicide research varies according to methodological and design differences and cultural dimensions [27]. In this study, the professionals agreed on the importance of understanding the complex symptoms of sleep and rest, stress and inner turmoil, family problems and alcohol use in suicidal behaviour.

### Importance for treatment

The assessments of treatment measures in CAP and DPC were generally quite similar. Psychologists and social workers considered psychotherapy most important for treatment of suicidal behaviour. The present study did not differentiate interventions according to the patients’ main problems. The indication for use of medication usually differs between patients with serious depression, personality disorders or crisis reactions. The same holds true for the use of ECT, which was considered to have little importance by both CAP and DPC professionals. However, the use of ECT is considered appropriate for a minority of the patients in these units.

Admission to a psychiatric ward was considered the second most important treatment for suicidal behaviour by both CAP and DPC professionals. DPC professionals agreed more with the use of medication than those in CAP, with no difference between professional groups. The reluctance in CAP to use medication might also be influenced by the on-going discussion on whether anti-depressants can increase suicidal ideation in adolescents [28]. Views on both causes of suicidal behaviour and appropriate treatment should be assessed preferably according to subgroups of patients in future studies: e.g. patients with psychoses, affective disorders, personality disorders and other subgroups.

### Treatment available

Neither CAP nor DPC professionals agreed that patients with suicidal behaviour are offered treatment and support equivalent to that offered to other patients with serious health problems such as heart disease. This may also reflect the tendency for priorities in health care to be based more on acceptable social values than on the severity of the medical condition. Suicidal behaviour often reflects a serious health condition requiring long-term follow-up and preferably continuity of care. Follow-up routines for patients with heart disease and cancer are organized generally more systematically than for suicidal behaviour, even though their mortality and morbidity rates are comparable.

In general, professionals in CAP were less satisfied than professionals in DPC with the length of follow-up and with opportunity for admission to a psychiatric ward when required.

The differences may indicate that professionals in CAP perceive treatment of suicidal behaviour to be more difficult than do their counterparts in DPC because they perceive that they receive limited support to do their work competently. Suicide is more prevalent in adult populations and suicidal behaviour in adolescent populations, the latter often being associated with relational problems [9].

### Generalizability

Psychiatric services in Norway are homogeneously organized. Our sample covered the four health regions in Oslo and the professional groups involved in treatment, with a 77% response rate. Accordingly, our findings could be generalized to other Norwegian CAP and DPC outpatient units. However, they cannot be generalized to psychiatrists and psychologists in private practice without further studying this population and, by extension, professionals in internal medicine, surgery, or general practice who treat patients with suicidal behaviours.

### Validity and reliability

The ATTS and USP have been validated in several previous studies [20]. Nevertheless, professionals are expected to have a positive attitude towards patients, including those with suicidal behaviour, in line with expectations and recommendations from the national guidelines [25]. Accordingly, there is a possibility that the responses are biased positively towards social desirability [29]. There is always a possibility of response bias in surveys like this. We consider that this is acceptable, as the response rate was satisfactory, all parts of the capital of Norway were covered and the answers were anonymous.

### Strengths and limitations

The strengths of our study include a representative sample of Oslo and a high response rate. Our study also takes into account the possible influences of the different CAP and DPC environments in which physicians and psychologists are treating patients with suicidal behaviour. It is likely that the findings from Oslo are also representative for other DPC and CAP professionals in Norway, as education and specialization and the general culture is rather uniform. Studies with greater samples are needed, however, to know whether the findings can also be generalized to subgroups of professionals with different religions or ethnic backgrounds.

We have no information on what training is most successful, which may be addressed in other prospective studies.

An evaluation of treatments available for patients with different diagnoses would be possible with a larger sample. Our finding of a minority of suicidal patients being treated with ECT was reflected in its consideration by professionals as a treatment of low relevance. It is more likely that more professionals would find ECT relevant if they had been asked about patients with severe depression and psychomotor retardation.

Future research should consider determining the subscales on theoretical grounds or by using Item Response Theory.

### Clinical implications

The study has some clinical implications. Professionals that were specialists or had gained supervision had somewhat more positive attitudes. This calls for more systematic focus on attitudes in training and supervision. The majority of the professionals had not gained supervision specifically on suicidal patients. In addition, it may be rewarding for the professionals to get confirmation that their attitudes are positive in this clinically challenging setting.

The relationship between supervision and training and a positive attitude towards suicidal patients’ calls for more systematic measures that ensure all professionals who treat patients with suicidal behaviour can access appropriate educational resources. Because suicidal ideation is associated with ambivalence towards life and death and hopelessness about the future, professionals must project an attitude of optimistic encouragement. Even though many patients with moderate suicidal intention may be treated in outpatient units, patients who become highly suicidal must have access to in-patient treatment. The professionals in CAP in particular were not satisfied with the current availability of in-patient treatment.

The professionals’ dissatisfaction with the duration of follow-up also indicates a need for measures that offer suicidal patients treatment comparable to the treatment of patients with other serious health problems. Suicidal behaviour mostly reflects serious and long-lasting mental health problems that need continuity of care and long-term follow-up.

## Conclusions

Health professionals in mental health outpatient units in Oslo reported a positive attitude towards patients with suicidal behaviour. They also confirmed a belief that suicide can be prevented. Only minor differences were found between equivalent professions in CAP and DPC. However, those with access to supervision or those who were specialists indicated a more positive attitude towards suicidal behaviours. Psychiatric disorders were considered the most common reason for suicidal behaviour and psychotherapy the most relevant treatment. The professionals indicated a shared understanding that patients with a condition of comparable severity such as heart disease received a more systematic follow-up.

## Consent

The professionals were written informed about the purpose of the study for publishing and participated voluntarily.

## Abbreviations

CAP: Child and Adolescent Psychiatry; DPC: District Psychiatric Centre; USP: Understanding of Suicidal Patient Scale; ATTS: Attitudes Towards Suicide; SOQ: Suicide Opinion Questionnaire; ECT: Electroconvulsive therapy; N,s*: Not significant.

## Competing interests

The authors declare that they have no competing interests.

## Authors’ contributions

ABN participated in research design, organized, collected and analysed the data and wrote the manuscript. TKG participated in research design, data analysis and supervised the work. ØE supervised the study from design to manuscript. All authors read and approved the final manuscript.

## Authors’ information

ABN: Works as a psychiatric nurse in consulting psychiatry in Diakonhjemmet Hospital and is associated with the Regional competence centre on violence, traumatic stress and suicide prevention, eastern region of Norway.

TKG: Works as a PhD candidate in the Department of Acute Medicine at Oslo University Hospital and cooperates with the Regional competence centre on violence, traumatic stress and suicide prevention, eastern region of Norway.

ØE is a consultant psychiatrist in the Department of Acute Medicine, Oslo University Hospital, Ulleval. He is also professor at the Institute of Basic Medical Science, Department of Behavioural Science in Medicine, Faculty of Medicine, University of Oslo, Norway.

## Pre-publication history

The pre-publication history for this paper can be accessed here:

http://www.biomedcentral.com/1471-244X/13/90/prepub
